# Breech Presentations

**Published:** 1878-01-20

**Authors:** J. E. Clark


### *BREECH PRESENTATIONS.*

By J. E. Clark, M.D.

The relative proportion of breech presentations to presentations of other parts of the fœtus, varies considerably, as reported from different institutions.

Scanzoni gives the number from the lying-in asylums of Prague and Wurzburg as about one in fifty-six. Grenser, in the report of the lying-in institute of Dresden for six years, one in sixty-six, while Ramsbotham, Jr., from the Maternity of London, estimates them as about one in thirty-five.

I have been unable to find any reliable statistics as to the proportion of still-born children in these presentations, but it is known to be large.

The progress of labor is much slower, both in the first and second stage, when the breech presents than it is when head presents. From the nature of the presenting part dilatation is not so readily accomplished, and the parts do not adapt themselves so readily to the pelvic cavity.

The breech is more liable to be arrested in its descent than the head. The arrest of the breech, especially in a primipara, becomes the occasion of great and protracted suffering to the mother, very probable death of the child, and a source of great anxiety to the physician. They are, in fact, formidable cases to treat, and the physician having seen one becomes very desirous to avoid another.

Inasmuch as we can never tell when we are going to have trouble in these cases, it is better to prevent the breech becoming arrested if possible.

The rule I have followed in my practice for many years now is, in all cases of breech presentations at full time, to bring down a foot. This allows complete control of the labor; we can hasten it as the exigencies of the case may require.

Dr. Robert Barnes, of London, adopted this mode of treatment in cases where the breech becomes arrested. Would it not be better to do the same thing earlier, and thus prevent hours and hours of intense agony to the mother and danger to the child?

I prefer to perform the operation before the first stage of labor is completed. It can be done then very easily, and without inflicting much suffering upon the mother. It is seldom necessary to give chloroform, though there is no objection to it if desired. After the foot is brought down the dilatation of the os uteri is more readily completed, and the duration of the labor much shorter.

There are some points as to the manner of performing the operation I would like to mention. The feet and legs occupy two different positions in these cases. In one, and the most common by far, the legs are flexed upon the thighs, which brings the feet very near the os uteri. In the other, the legs are extended, carrying the feet near the fundus of the uterus, by the side of the head. Of course, these last are the most difficult to manage, and rarely fail to give trouble if left to themselves. I have adopted the following rules:

1. In introducing the hand into the uterus, use great gentleness with firmness, and always support the fundus with the unoccupied hand.
2. Introduce the hand, the palmar surface of which will pass readily along the posterior aspect of the thigh of fœtus.
3. Choose the foot most anterior.
4. Never bring down but one foot—reasons obvious. It leaves protection for cord, and gives bulk for dilatation.
5. Do not hasten the passage of the hips through the pelvis Secure all the dilatation possible.
6. Guide the rotation of the child in its descent, so that the abdomen is posterior in relation to the mother.

I have said nothing in regard to the diagnosis in these cases, because the points of diagnosis are well known, and so easily made out, that a mistake can only occur through great and inexcusable carelessness.—*Society County Kings.*

